# The potential for minimally invasive intracerebral hemorrhage evacuation in routine healthcare: applicability of the ENRICH trial criteria to an unselected cohort

**DOI:** 10.3389/fstro.2024.1403812

**Published:** 2024-05-17

**Authors:** Trine Apostolaki-Hansson, Amir Hillal, Nathanael Göransson, Björn M. Hansen, Bo Norrving, Birgitta Ramgren, Johan Wassélius, Teresa Ullberg

**Affiliations:** ^1^Department of Clinical Sciences Lund, Lund University, Lund, Sweden; ^2^Department of Neurology, Skåne University Hospital, Malmö, Lund, Sweden; ^3^Medical Imaging and Physiology, Skåne University Hospital, Lund, Sweden; ^4^Department of Neurosurgery, Skåne University Hospital, Lund, Sweden

**Keywords:** intracerebral hemorrhage (ICH), minimally invasive (MI), minimally invasive surgery (MIS), computed tomography, epidemiology, population-wide, eligibility assessment, neurosurgical procedures

## Abstract

**Objective:**

Following the favorable outcomes demonstrated by the Early MiNimally-invasive Removal of IntraCerebral Hemorrhage (ENRICH) trial in supratentorial intracerebral hemorrhage (ICH) patients treated with minimally invasive surgery (MIS), and considering the increasing interest in MIS, we aimed to assess the potential eligibility rate for ICH patients in Sweden.

**Methods:**

All patients with spontaneous ICH in the Swedish Stroke Register (RS) during 2017–2020 in Skane county (1.37 million) were assessed. Baseline imaging was used for radiological characterization. Clinical data were obtained from RS. MIS eligibility in the total ICH population meeting ENRICH criteria were estimated and extrapolated to the Swedish population (10.5 million).

**Results:**

Of 1,314 ICH patients, 5.9% met the ENRICH criteria for MIS (ICH volume 30–80 ml). Considering the ENRICH trial results indicating the effectiveness of MIS was mainly attributable to intervention for lobar hemorrhages, we determined that 2.8% of our ICH cohort in Sweden would be eligible for MIS. The estimated rate of neurosurgery for ICH could increase from the current 1.46–1.90 patients/100,000 population/year (in absolute numbers from 154 to 200 interventions out of 2,400 ICHs in Sweden annually).

**Conclusions:**

We show that 2.8% of the Skane ICH population would be eligible for MIS if ENRICH criteria are employed for patients with lobar ICH, corresponding to a 29% increase of current surgical rates for ICH in Sweden. As MIS for ICH is not yet standard practice in Sweden, consideration for its implementation within the neurosurgical organization becomes essential to accommodate the anticipated increase in patient demand.

## Introduction

Intracerebral hemorrhage (ICH) amount to 10–30% of all strokes, but accounts for half of the mortality and morbidity related to stroke (Chen et al., 2020) including a 30-day mortality rate up to 40% (Qureshi et al., 2001; Collaborators GBDS, 2019). According to current guidelines, minimally invasive surgical evacuation of supratentorial hemorrhage can decrease mortality but effects on functional outcome are uncertain (Greenberg et al., 2022). The minimally invasive surgery with thrombolysis in intracerebral hemorrhage evacuation (MISTIE III) trial showed that minimally invasive surgery (MIS) lowered mortality, but did not improve functional outcome 1-year post-surgery (Hanley et al., 2019). A subsequent sub-study showed that outcome was highly associated with the surgical result of hematoma evacuation, more specifically with a residual hematoma volume of ≤ 15 ml, which was only achieved in 58% of surgically treated patients in the MISTIE III trial (Awad et al., 2019).

Recently, the Early MiNimally-invasive Removal of IntraCerebral Hemorrhage (ENRICH) trial showed improved mortality and functional outcome for patients with a supratentorial ICH volume of 30–80 ml treated by MIS compared to standard care (Pradilla et al., 2024). The effect of surgery was mainly attributable to intervention for lobar hemorrhages. If successfully replicated, and if resources permit, this intervention could be implemented on a larger scale. However, the magnitude of translation of the study's patient selection to a nationwide ICH population is unknown.

This study aims to estimate the proportion and volume of patients eligible for early MIS evacuation based on ENRICH criteria in the ICH population in Sweden using data from the Swedish Stroke Register (Riksstroke) combined with radiological data from the Skane hospital region (Hillal et al., 2023).

## Methods

### Patient population and database

All patients (age ≥18) in Skane county (1.37 million inhabitants, 13.3% of the Swedish population 2017–2020) registered with spontaneous ICH in Riksstroke 1/1/2017–31/12/2020 were included in this study. Riksstroke had >90% coverage of Swedish ICH patients during the study period (Riksstroke, 2020). Data on mortality status were obtained from the Swedish Cause of Death Register, with a >98% coverage (Register TSCoD, 2023). Patients without available non-contrast computed tomography (NCCT) images in the regional picture archiving and communication system (PACS) were excluded from the study (*n* = 10). The Riksstroke database was radiologically validated to clinical data, and cases that were not defined as spontaneous ICH were excluded from the analysis; hemorrhagic transformation of ischemic stroke, aneurysmal bleeds related to subarachnoid hemorrhage (SAH)/ICH, tumor bleeds, and traumatic ICH/subdural hemorrhage/SAH. Intracerebral hemorrhage caused by vascular malformations was defined as spontaneous ICH but was excluded from this analysis in line with the ENRICH criteria.

In this study, patients matching the ENRICH study cohort were 18–80 years of age, pre-stroke independent, presented <24 h from symptom onset with an altered level of consciousness (LOC), and had a supratentorial spontaneous ICH without an underlying vascular malformation. These patients additionally had an ICH volume of 30–80 ml on baseline non-contrast computed tomography (NCCT) measured by manual segmentation. Given that the ENRICH trial indicated that patients with lobar ICH receiving both surgical and medical treatments exhibited improved weighted disability scores compared to medical treatment alone, we opted to assess the proportion of patients within our study population with lobar hemorrhage who would meet the criteria for MIS. Results were extrapolated to the regional and total Swedish population using official census data (Sweden, 2023).

As several ongoing randomized clinical trials recruiting supratentorial ICH patients for MIS compared to standard care include a intracerebral hemorrhage volume cutoff value of ≥20 ml (EMINENT-ICH NCT05681988, DIST NCT05460793, HEALME NCT05138341, EVACUATE NCT04434807) and a broader inclusion of GCS (EMINENT-ICH NCT05681988, DIST NCT05460793, EVACUATE NCT04434807), different scenarios were explored to expand the proportion of patients eligible for MIS in patients with lobar ICH.

### Variables

Data on baseline variables were extracted from Riksstroke and included patient demographics, vascular risk factors, medications prior to ICH, and use of anticoagulant reversal and neurosurgery. Riksstroke uses the Reaction Level Scale (RLS-85) to assess level of consciousness, an 8 grade level of consciousness assessment tool that correlates well with the Glasgow Coma Scale (GCS; Starmark et al., 1988), including categories of alert (RLS 1 corresponding to GCS 14–15), drowsy (RLS 2–3 i.e., GCS 9–13), and comatose (RLS 4–8 i.e., GCS 3–8).

The pre-stroke mRS status is not available in Riksstroke. Instead, variables pertaining to the patient's functional status prior to ICH were used to estimate pre-stroke functional status. Pre-stroke independency was characterized by patients who did not require homecare, had the ability to dress and attend to toileting autonomously, and exhibited autonomous indoor and outdoor mobility. Conversely, pre-stroke dependency was defined as patients receiving homecare or who were residing in an assisted living facility or comparable institutions, and/or who relied on assistance for dressing, toileting, and/or mobility.

Reading of NCCT images was performed by two independent radiologists. Radiological variables included the following: time of first imaging, volume of ICH and intraventricular hemorrhage, location (supra-/infratentorial, deep/lobar), intraventricular extension, and the presence of vascular malformations. The radiological database was validated and cases incorrectly coded as spontaneous ICH were excluded prior to this study.

### Statistics

SPSS v28 was used for all analyses. Data were presented as simple frequencies and medians with corresponding interquartile ranges. Proportions were compared using *x*^2^-test.

## Results

### Baseline data

Baseline characteristics are shown for the entire ICH cohort (*n* = 1,314) and for those matching the ENRICH study cohort, with variables for volume and level of consciousness defined within the table (*n* = 554; Table 1). The median ICH volume was 16 ml in both groups. Mean age was 69 in the cohort matching the ENRICH population compared to 76 years in the entire ICH cohort. The proportion of female patients was 36.5% in the ENRICH matched cohort, and 44.4% in the total ICH cohort. The proportion of patients treated with neurosurgery in the entire cohort was stable during 2017–2020; correspondingly 4.6, 6.1, 7.1, and 6.6% (*p* = 0.673).

**Table 1:** Baseline data and outcome characteristics for the entire ICH population as well as the population with supratentorial ICH, age 18–80, pre-stroke independent comparable to the ENRICH population.

**Variables**	**All ICH *n* = 1,314**	**Supratentorial ICH, age 18–80, pre-stroke independent (*n* = 554)**
**Demographics**		
Median age (IQR)	76 (67–84)	69 (61–75)
Female sex	44.4% (583)	36.5% (202)
Pre-stroke independent	67.6% (888)	100% (554)
**Vascular risk factors**		
Hypertension	60.4% (794)	50.2% (278)
Diabetes	19.1% (251)	16.2% (90)
Atrial fibrillation	27.8% (365)	16.2% (90)
Previous stroke	25.4% (334)	17.0% (94)
**Antithrombotic medication at onset**		
Antiplatelet	25.3% (333)	23.1% (128)
Warfarin	14.3% (188)	8.3% (46)
DOAC	13.4% (176)	7.9% (44)
Reversal of OAC (*n* = 364)	66.8% (243/364)	76.7% (69/90)
**Clinical characteristics**		
**Level of consciousness**		
*Alert*	54.0% (710)	65.9% (365)
*Drowsy*	26.1% (343)	21.3% (118)
*Comatose*	19.2% (252)	12.3% (68)
Neurosurgical intervention	6.1% (80)	10.1% (56)
**Radiological characteristics**		
**Location**		
Supratentorial	81.1% (1,088)	100% (554)
Infratentorial	12.5% (167)	–
Both	3.1% (42)	–
IVH only	3.3% (44)	–
Lobar	39.2% (526)	44.2% (245)
Deep	44.7 (600)	55.8% (309)
Both	0.3% (4)	–
IVH extension	44.7% (599)	34.3% (190)
**Hemorrhage volume**		
Total volume median (IQR)	16 (4–45)	16 (4–40)
Parenchymal volume (IQR) in patients with IVH extension	19.5 (7–46) (*n* = 599)	25 (8–46) (*n* = 190)
**Outcomes**		
30-day mortality	30.1% (396)	15.7% (87)
30-day mortality for patients treated neurosurgically	5.0% (4/80)	5.4% (3/56)

DOAC, direct oral anticoagulant; ICH, intracerebral hemorrhage; IQR, interquartile range; IVH, intraventricular hemorrhage; OAC, oral anticoagulant; ml, milliliters.

**Figure 1:**
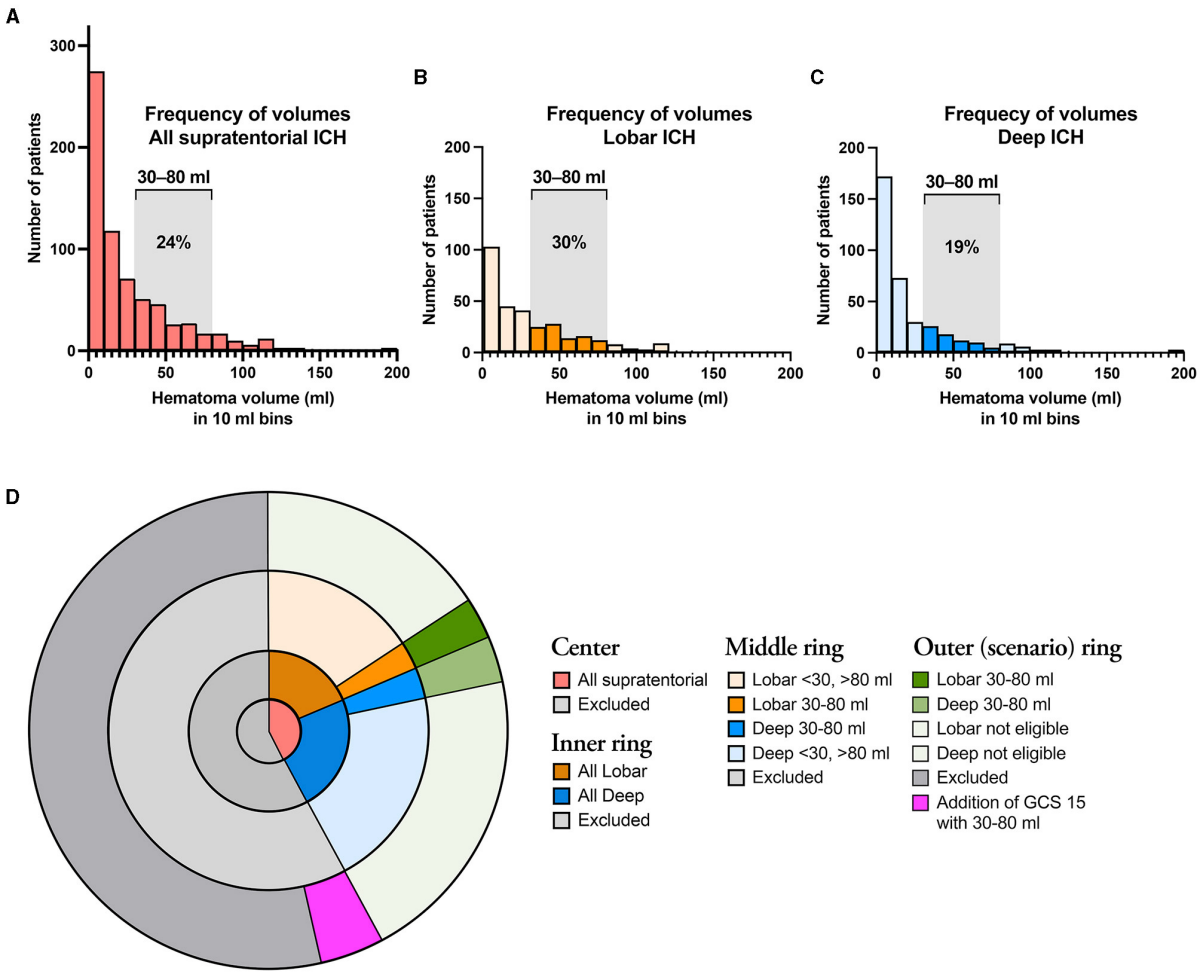
**(A)** Illustrates the distribution of all supratentorial ICH cases according to hematoma volume, and separately for lobar **(B)** and deep **(C)** ICH. The assessed ICH volumes 30–80 ml are indicated by saturated colors in the latter two graphs. **(D)** Illustrates the proportions of ICH patients that fulfill the ENRICH criteria (30–80 ml) for deep and lobar ICH. The center shows the proportions of all supratentorial ICH that fulfill the non-volumetric ENRICH criteria (red), as well as excluded patients (dark gray); the Inner ring shows the proportions of lobar (brown) and deep (blue) supratentorial ICH; the Middle ring illustrates the proportions of lobar (brown) and deep (blue) supratentorial ICH according to hemorrhage volume. The outermost ring illustrates eligibility according to the ENRICH criteria supratentorial ICH of 30–80 ml (dark green) as well as to the extrapolated scenario of addition of supratentorial ICH of 30–80 ml in patients with GCS 14–15.

Figure 1A illustrates the distribution of supratentorial ICH volumes (*n* = 554), with separate illustrations for lobar (*n* = 245; Figure 1B) and deep (*n* = 309; Figure 1C) supratentorial ICH. In patients matching the ENRICH study cohort, hemorrhage volume was <30 ml in 67.0% (371/554) of cases and >80 ml in 8.7% (48/554) of cases. Supplementary Figure 1 shows patients excluded from analysis. Patients excluded from analysis were >80 years old, pre-stroke dependent, had an isolated IVH, and/or an infratentorial ICH.

### Eligibility for MIS using different scenarios

Based on the ENRICH criteria (ICH volume 30–80 ml), the proportion of patients eligible for MIS in the entire ICH population was 5.9% [78/1 314; 37 lobar (2.8%), 41 deep (3.1%); Figure 1B]. Considering only patients with lobar ICH who exclusively met the ENRICH criteria (Table 2), the proportion eligible for MIS within the entire ICH cohort was 2.8% (*n* = 37/1 314). Baseline characteristics are presented in the table.

**Table 2:** Baseline data and outcome characteristics for the total supratentorial intracerebral hemorrhage population that met the ENRICH criteria including both deep and lobar hemorrhages (left column), and for patients with lobar intracerebral hemorrhage meeting ENRICH criteria (right column), age 18–80, pre-stroke independent, intracerebral hemorrhage volume 30–80 ml, with altered level of consciousness.

**Variables**	**Deep and Lobar ENRICH-matched Volume 30–80 Altered LOC (*n* = 78)**	**Lobar only ENRICH-matched Volume 30–80 Altered LOC (*n* = 37)**
**Demographics**		
Median age (IQR)	69 (61–75)	68 (61–75)
Female sex	44.9% (35)	51.4% (19)
Pre-stroke independent	100% (78)	100% (37)
**Vascular risk factors**		
Hypertension	55.1% (43)	48.6% (18)
Diabetes	17.9% (14)	13.5% (5)
Atrial fibrillation	21.8% (17)	18.9% (7)
Previous stroke	25.6% (20)	16.2% (6)
**Antithrombotic medication at onset**		
Antiplatelet	29.5% (23)	29.7% (11)
VKA	10.3% (8)	10.8% (4)
DOAC	10.3% (8)	10.8% (4)
Reversal of OAC	81.3% (13/16)	87.5% (7/8)
**Clinical characteristics**		
**Level of consciousness**		
Alert	–	–
Drowsy	59.0% (46)	73.0% (27)
Comatose	41.0% (32)	27.0% (10)
**Radiological characteristics**		
**Location**		
Supratentorial	100% (78)	100% (37)
Infratentorial	–	–
Both	–	–
IVH only	–	–
Lobar	47.4% (37)	100% (37)
Deep	52.6% (41)	–
Both	–	–
IVH extension	67.9% (53)	59.5% (22)
**Hemorrhage volume**		
Total volume median (IQR)	50.5 (39–65)	55 (39–65)
Parenchymal volume (IQR) in patients with IVH extension	37 (25–49) (*n* = 53)	58 (45–66) (*n* = 22)
Neurosurgical intervention	29.5% (23)	35.1% (13)
**Outcomes**		
30-day mortality	41.0% (32)	29.7% (11)
30-day mortality for patients treated neurosurgically	4.3% (1/23)	7.7% (1/13)

DOAC, direct oral anticoagulant; ICH, intracerebral hemorrhage; IQR, interquartile range; IVH, intraventricular hemorrhage; OAC, oral anticoagulant; ml, milliliters; VKA, Vitamin K antagonist.

As several ongoing RCTs investigating MIS vs. standard care in patients with supratentorial ICH, including patients with an ICH volume of ≥ 20 ml and patients presenting with altered or alert LOC, are currently undergoing or are planned in the near future, we opted to extend the lower limit of the ICH volume criteria in our study to include smaller lobar ICH volumes ranging from 20 to 80 ml. As a result, the proportion of patients eligible for MIS increased to 3.2% (42/1 314). If the inclusion criteria regarding level of consciousness were extended to include patients presenting with a GCS score of 14–15, the proportion of patients eligible for MIS would increase to 5.86% (77/1 314) in patients with an ICH volume of 30–80 ml, and to 8.14% (107/1 314) in patients with an ICH volume of 20–80 ml (Figure 1D). Baseline characteristics are demonstrated in Supplementary Table 1 for the different scenarios in patients eligible for MIS with lobar ICH.

Current rates of neurosurgery and 30-day all-cause mortality in the patient cohort, between 2017 and 2020, are shown in Tables 1, 2. Less than one third of patients that fall under the ENRICH criteria are currently treated with neurosurgery (29.5%; *n* = 23/78). Patients treated with neurosurgery had an overall 30-day mortality rate of 4% compared to 30.1% for the whole ICH population. Among patients with lobar ICH who met the ENRICH criteria, neurosurgery was performed in 35.1% of cases (*n* = 13/37), with a corresponding 30-day mortality rate of 7.7% in these patients.

### Extrapolation of patient volumes

The crude ICH rate was 24.0/100 000/year in our observed population (Skane hospital region; *n* = 1,368,504) of which 1.42 patients/100,000/year were estimated to be eligible for MIS using the ENRICH criteria for any supratentorial ICH, of which 0.68 patients/100,000/year with lobar ICH would be eligible.

In the Skane region, 0.44 patients/100,000/year would be eligible for MIS in addition to the 1.46 patients/100,000/year that currently undergo neurosurgical treatment; corresponding to an increase with a factor 1.30. In absolute numbers, concerning the overall approximate annual ICH population (*n* = 2,400), an estimated 46 additional patients per year could be eligible for MIS nationwide if applying the ENRICH criteria for patients with lobar ICH. This is in addition to the estimated 154 ICH patients that are currently admitted to neurosurgery.

If using the extended ENRICH criteria (lobar ICH volume 20–80 ml and all levels of consciousness), 1.95 patients/100,000/year were estimated to be eligible for MIS. In the Skane region, 1.56 patients/100,000/year would be eligible for MIS in addition to the 1.46 patients/100,000/year that currently undergo neurosurgical treatment; corresponding to an increase with a factor 2.07 if using the extended ENRICH criteria.

## Discussion

We assessed the scale of the implementation of the ENRICH criteria in the Skane hospital region by modeling the proportion of eligible ICH patients based on data from Riksstroke combined with radiological data from the regional PACS to ensure >90% coverage of the entire ICH population (Hillal et al., 2023). Based on these criteria, we determined that 5.9% of the entire ICH patient cohort in the Skane hospital region would be eligible for MIS during the study period. Considering that results from the ENRICH trial indicated that the effect of surgery was mainly attributable to intervention for lobar hemorrhages, we further determined that 2.8% of our ICH patient cohort would be eligible for MIS.

Hematoma evacuation decreases mortality in patients with elevated intracerebral pressure due to mass effect, but patients with smaller hematomas (20–30 ml) and those without altered consciousness could potentially benefit from MIS through hematoma clearance that might possibly result in an improved functional outcome. The ENRICH trial has shown benefit in patient outcomes when hematoma clearance is considered for patients with lobar ICH meeting the inclusion criteria. While no current evidence exists regarding the following, it is notable that several ongoing RCTs addressing MIS for supratentorial ICH include patients with smaller hematoma volumes (≥20 ml) and a broader inclusion criteria of patients with an alert LOC at admission. We therefore estimated the proportion of patients with lobar ICH eligible for MIS based on distinct scenarios including different ICH volume cut-offs and level of consciousness in order to determine how these potential extensions of the ENRICH criteria would translate to the entire ICH population. Results show that such extensions would have a substantial increase in the proportion of ICH patients eligible for MIS, extending to 8.14% of the ICH population if including patients presenting alert with a hemorrhage volume of 20–80 ml. In absolute numbers, this could lead to a 100% increase of current rates of neurosurgery in Swedish practice, from 1.46 to 3.02 ICH patients receiving any neurosurgical intervention/100,000 inhabitants per year.

Any neurosurgical intervention was employed in 6.1% of the entire population during 2017–2020, of which one fourth of patients had an infratentorial ICH. The variable *any neurosurgical intervention* is not limited to patients receiving hematoma evacuation by craniotomy but can also include other interventions (such as external ventricular drainage or intracranial pressure monitoring) as Riksstroke registers all surgical interventions into a single variable. Among patients fulfilling the ENRICH criteria with lobar ICH, 35.1% received any neurosurgical treatment. Therefore, the implementation of MIS for patients with lobar ICH according to the ENRICH criteria would result in an increase in neurosurgical candidates, consequently increasing neurosurgical capacity. The estimated rate of neurosurgery for ICH could increase from the current 1.46–1.90 patients/100,000 population/year. In absolute numbers, this would mean an increase from 154 to 200 interventions annually out of 2,400 ICHs in Sweden. Given that MIS for ICH patients is not currently a standard procedure in Sweden, it may become essential to prioritize training initiatives and effectively allocate resources within the neurosurgical organization to accommodate this anticipated increase in patient demand.

The current low proportion of patients receiving any type of neurosurgical intervention emphasizes that surgical hematoma evacuation by craniotomy could potentially be widely replaced by the MIS method. In 2021, 8% of all ICH patients in Sweden (*n* =2,400) were candidates for *any neurosurgical intervention*. The corresponding proportion for neurosurgery in ICH patients in the Skane Region was 6%. For comparison with ischemic stroke in Sweden, endovascular treatment using mechanical thrombectomy is currently performed in 9% of ischemic stroke cases, corresponding to a volume of ~1,200 treatments per year, and 14% are treated with intravenous thrombolysis (Register, 2023). However, due to the higher incidence of ischemic stroke, patient volumes are ~7-fold larger compared to ICH patients.

A significant proportion of patients meeting the ENRICH criteria were taking antithrombotic medications at hospital presentation, such as an antiplatelet or oral anticoagulant drug, constituting 50% of cases. Patients fulfilling the ENRICH criteria had altered LOC at presentation, and a notably higher mortality rate compared to those meeting the extended ENRICH criteria (ICH volume 20–80 ml, with all LOC), at 29.7 vs. 15.0%, respectively. Although the mortality rates presented are unadjusted, the observed difference may be attributed in part to the larger proportion of patients on antithrombotic medications prior to ICH in the strict ENRICH criteria cohort. Both the ENRICH trial and the MISTIE III trial did not explicitly exclude patients taking oral anticoagulant drugs when considering eligibility for MIS. However, the safety of MIS among ICH patients using oral anticoagulant (OAC) drugs has not been specifically evaluated in sub-studies presumably due to the novelty of the procedure. The availability and the use of direct antidotes for patients taking direct oral anticoagulants (DOAC) prior to ICH, and prothrombin complex concentrate for patients on vitamin K antagonists (VKA), are important treatment strategies in the management of ICH and are essential for ensuring the safety and efficacy of MIS in patients with OAC related ICH.

### Limitations

Firstly, all details on exclusion criteria present in the ENRICH trial were not available in our data, the proportion of MIS cases could therefore be slightly overestimated. Patients with Moyamoya disease, venous sinus thrombosis, those with recurrence of a recent ICH (≤1 year), and those with thalamic hemorrhages were not excluded in our patient cohort, the proportion of these cases is, however, estimated to be low. There was a lower than expected proportion of patients that were diagnosed with vascular malformations (*n* = 27) in the total ICH population, this is presumed to be due to few patients undergoing computed tomography angiography (CTA) or magnetic resonance imaging (MRI) in their diagnostic workup. Secondly, other exclusion criteria present in the ENRICH trial including data on NIHSS score and other patient and laboratory characteristics (for e.g., end-stage liver/renal disease, platelet count, pupil size, pregnancy, mechanical heart valves, life-expectancy, withdrawal of care orders, etc.) were unidentifiable in our patient cohort since Riksstroke does not account for these variables. Data concerning neurological deterioration after presentation were also unavailable, which could lead to an underestimation of the true proportion of patients that were eligible for MIS in the study cohort. Thirdly, data on the use of rapidly reversible OACs were available, however it was not applied as an exclusion criterium in our analysis since the direct antidote for factor Xa inhibitor reversal is now available and should not be an exclusion criterion for neurosurgery in the future. Lastly, the ENRICH criteria exclude GCS ≤ 4, we were unable to exclude this patient population in our analyses since Swedish patients are classified as alert, drowsy, or comatose at first presentation according to the Reaction Level Scale (RLS-85) commonly used in Sweden.

## Conclusions

In conclusion, we determined that a conservative estimate of 2.8% of the entire ICH population would be eligible for MIS if implementing the ENRICH criteria for lobar ICH in routine clinical care in Sweden. This would consequently result in ~30% increase in neurosurgical interventions per year for patients with lobar ICH. Considering ongoing RCTs, extending the lower limit of ICH volume to 20–80 ml and including patients with a GCS score of 14–15 at admission, in cases of lobar ICH, would result in 8.14% eligibility for MIS.

## Data availability statement

An anonymized dataset, supporting the conclusions of this article may be provided upon reasonable request.

## Ethics statement

The studies involving humans were approved by Swedish Ethical Review Authority (#2020–06800). The studies were conducted in accordance with the local legislation and institutional requirements. Written informed consent for participation was not required from the participants or the participants' legal guardians/next of kin in accordance with the national legislation and institutional requirements.

## Author contributions

TA-H: Conceptualization, Data curation, Formal analysis, Investigation, Methodology, Validation, Writing—original draft, Writing—review & editing. AH: Conceptualization, Data curation, Formal analysis, Investigation, Methodology, Validation, Writing—original draft, Writing—review & editing. NG: Investigation, Writing—original draft, Writing—review & editing. BH: Conceptualization, Data curation, Formal analysis, Investigation, Methodology, Software, Supervision, Validation, Writing—original draft, Writing—review & editing. BN: Conceptualization, Methodology, Supervision, Writing—original draft, Writing—review & editing. BR: Data curation, Investigation, Writing—original draft, Writing—review & editing. JW: Conceptualization, Funding acquisition, Investigation, Methodology, Project administration, Resources, Software, Supervision, Visualization, Writing—original draft, Writing—review & editing. TU: Conceptualization, Data curation, Formal analysis, Investigation, Methodology, Project administration, Software, Supervision, Validation, Writing—original draft, Writing—review & editing.
