# Supplementary material for: The potential for minimally invasive intracerebral hemorrhage evacuation in routine healthcare: applicability of the ENRICH trial criteria to an unselected cohort
Source: Front Stroke. 2024 May 17;3:1403812. doi: 10.3389/fstro.2024.1403812 (PMC12802725; doi:10.3389/fstro.2024.1403812)
Supplement: Supplementary file 1 [file Table_1.docx]

Supplemental Table 1

| Variables | ENRICH-matched  Volume 30–80  Altered LOC  (n=37) | Scenario 1  Volume 20–80  Altered LOC  (n=42) | Scenario 2  Volume 30–80  All LOC  (n=77) | Scenario 3  Volume 20–80  All LOC  (n=107) |
| --- | --- | --- | --- | --- |
| Demographics | | | | |
| Median age (IQR) | 68 (61–75) | 67 (61–74) | 71 (63–76) | 71 (64.5–76) |
| Female sex | 51.4% (19) | 47.6% (20) | 46.8% (36) | 39.3% (42) |
| Pre-stroke independent | 100% (37) | 100% (42) | 100% (77) | 100% (107) |
| Vascular risk factors | | | | |
| Hypertension | 48.6% (18) | 45.2% (22) | 53.2% (41) | 48.6% (52) |
| Diabetes | 13.5% (5) | 11.9% (5) | 6.5% (5) | 7.5% (8) |
| Atrial fibrillation | 18.9% (7) | 19.0% (8) | 15.6% (12) | 16.8% (18) |
| Previous stroke | 16.2% (6) | 14.3% (6) | 14.3% (11) | 17.8% (19) |
| Antithrombotic medication at onset | | | | |
| Antiplatelet | 29.7% (11) | 26.2% (11) | 31.2% (24) | 28.0% (30) |
| VKA | 10.8% (4) | 11.9% (5) | 11.7% (9) | 12.1% (13) |
| DOAC | 10.8% (4) | 9.5% (4) | 7.8% (6) | 6.5% (7) |
| Reversal of OAC | 87.5% (7/8) | 88.9% (8/9) | 86.7% (13/15) | 90.0% (18/20) |
| Clinical characteristics | | | | |
| Level of consciousness | | | | |
| Alert | – | – | 50.6% (39) | 59.8% (64) |
| Drowsy | 73.0% (27) | 76.2% (32) | 35.1% (27) | 29.9% (32) |
| Comatose | 27.0% (10) | 23.8% (10) | 13.0% (10) | 9.3% (10) |
| Radiological characteristics | | | | |
| Location | | | | |
| Supratentorial | 100% (37) | 100% (42) | 100% (77) | 100% (107) |
| Infratentorial | – | – | – | – |
| Both | – | – | – | – |
| IVH only | – | – | – | – |
| Lobar | 100% (37) | 100% (42) | 100% (77) | 100% (107) |
| Deep | – | – | – | – |
| Both | – | – | – | – |
| IVH extension | 59.5% (22) | 52.4% (22) | 37.7% (29) | 32.7% (35) |
| Hemorrhage volume | | | | |
| Total volume median (IQR) | 55 (39–65) | 49.5 (33–62) | 45 (36–57) | 39 (28.5–50.5) |
| Parenchymal volume (IQR) in patients with IVH extension | 58 (45–66) (n=22) | 58 (45–66) (n=22) | 56 (45–65) (n=29) | 50 (31–62) (n=35) |
| Neurosurgical intervention | 35.1% (13) | 31.0% (13) | 27.3% (21) | 20.6% (22) |
| Outcomes | | | | |
| 30-day mortality | 29.7% (11) | 28.6% (12) | 18.2% (14) | 15.0% (16) |
| 30-day mortality for patients treated neurosurgically | 7.7% (1/13) | 7.7% (1/13) | 4.8% (1/21) | 4.5% (1/22) |
| Abbreviations: DOAC = direct oral anticoagulant, ICH = intracerebral hemorrhage, IQR = interquartile range, IVH = intraventricular hemorrhage, OAC = oral anticoagulant, ml = milliliters, VKA = Vitamin K antagonist. | | | | |

Supplemental table 1. Baseline data and outcome characteristics for the lobar intracerebral hemorrhage population that met the ENRICH criteria: age 18–80, pre-stroke independent. Stratified according to different ENRICH criteria scenarios.
