# Supplementary material for: The potential for minimally invasive intracerebral hemorrhage evacuation in routine healthcare: applicability of the ENRICH trial criteria to an unselected cohort
Source: Front Stroke. 2024 May 17;3:1403812. doi: 10.3389/fstro.2024.1403812 (PMC12802725; doi:10.3389/fstro.2024.1403812)
Supplement: Supplementary file 2 [file Data_Sheet_1.docx]

Supplemental Figure 1


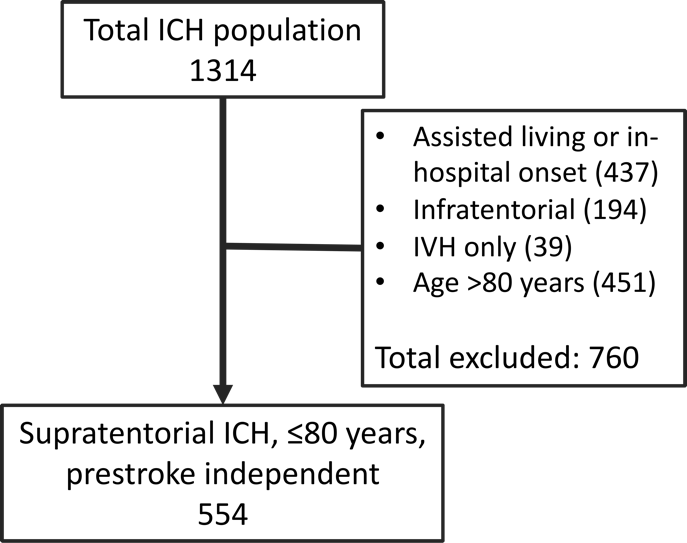


Supplemental Figure 1

Flow chart showing the criteria used to reduce the entire ICH population of 1314 patients to the final study population of 554 pre-stroke independent patients up to 80 years of age with supratentorial ICH.
